# Association Between Cognitive Function and the Autonomic Nervous System by Photoplethysmography

**DOI:** 10.3390/bioengineering11111099

**Published:** 2024-11-01

**Authors:** Jaewook Jin, Kahye Kim, KunHo Lee, Jeong-Woo Seo, Jaeuk U. Kim

**Affiliations:** 1 Digital Health Research Division, Korea Institute of Oriental Medicine, Daejeon 34504, Republic of Korea; jinjinjara98@kiom.re.kr (J.J.); kkh2@kiom.re.kr (K.K.); 2 Korean Convergence Medical Science, University of Science and Technology, Daejeon 34113, Republic of Korea; 3 Gwangju Alzheimer’s Disease and Related Dementias (GARD) Cohort Research Center, Chosun University, Gwangju 61452, Republic of Korea; leekho@chosun.ac.kr; 4 Department of Biomedical Science, Chosun University, Gwangju 61452, Republic of Korea; 5 Dementia Research Group, Korea Brain Research Institute, Daegu 41062, Republic of Korea

**Keywords:** photoplethysmography, pulse rate variability, Seoul neuropsychological screening battery, cognitive function, autonomic nervous system

## Abstract

This study explored the relationship between cognitive function and the autonomic nervous system by categorizing participants into two groups based on their cognitive function scores in each domain of the SNSB-D: a High Cognitive Performance (HCP) group and a Low Cognitive Performance (LCP) group. We analyzed the Pulse Rate Variability (PRV) parameters for each group. Photoplethysmography (PPG) data were collected and processed to remove noise, and the PRV parameters in the time and frequency domains were extracted. To minimize the impact of age and years of education on the PRV parameters, we performed an adjusted analysis using a Generalized Linear Model (GLM). The analysis revealed that the autonomic nervous system, particularly the parasympathetic nervous system, was more activated in the LCP group compared to the HCP group. This finding suggests that in individuals with low cognitive function, the sympathetic nerves in the autonomic nervous system are less activated, so the parasympathetic nerves are relatively more activated. This study investigated the correlation between cognitive function and PRV parameters, highlighting the potential use of these parameters as indicators for the early diagnosis and classification of cognitive decline.

## 1. Introduction

In the 21st century, the issues related to aging populations and declining birth rates have rapidly intensified, becoming a significant challenge to global societies. The increasing proportion of elderly individuals has profound implications, particularly for health care systems, which are under mounting pressure to address the complex health needs of older adults. Among the various health concerns, cognitive decline and cardiovascular dysfunction are especially problematic as they are closely linked to aging and can drastically reduce the quality of life of the elderly [1,2,3]. As such, there is a growing need for more sophisticated and proactive approaches to prevent and manage these age-related health problems.

To assess cognitive function, several standardized tools have been developed and widely used in clinical settings. Notably, the Seoul Neuropsychological Screening Battery (SNSB) is commonly utilized in South Korea for evaluating cognitive impairment, and the Mini-Mental State Examination (MMSE) is used more widely for short-form screening purposes [4]. The SNSB is a comprehensive neuropsychological test battery designed to assess various cognitive domains, including attention, language and related functions, visuospatial function, memory, and frontal/executive function [5]. It provides a detailed profile of cognitive abilities, allowing clinicians to diagnose conditions like mild cognitive impairment (MCI) and different types of dementia. The SNSB includes both basic screening tests, such as the MMSE, and more in-depth assessments tailored to detect cognitive changes across multiple domains [4,5]. While these tools are highly effective in providing a broad overview of cognitive health, they primarily rely on subjective responses and observed behaviors, and do not incorporate physiological data that may also reflect cognitive status [6].

Recent advances in biomedical technology have enabled researchers to explore the relationship between cognitive function and various physiological signals. Numerous studies have employed biosignals such as electroencephalography (EEG), photoplethysmography (PPG), electrocardiography (ECG), and functional magnetic resonance imaging (fMRI) to gain a deeper understanding of how cognitive function correlates with underlying physiological processes [7,8,9].

Among these methods, PPG has emerged as a promising tool due to its non-invasive nature, ease of use, and capacity to monitor cardiovascular function continuously. PPG is an optical technique that measures changes in blood volume in the microvascular bed of tissue using a light source and a photodetector. When light is emitted into the skin, it is either absorbed or reflected by the blood flowing through the tissues. The amount of light absorbed by the blood varies with the pulsatile changes in blood volume caused by the heartbeat, allowing the PPG device to capture these changes and produce a signal corresponding to the cardiovascular pulse wave. This technique is advantageous for its simplicity, affordability, and ability to provide the real-time monitoring of heart rate and pulse rate variability (PRV), which is derived from PPG signals and reflects autonomic nervous system activity [10].

Recent findings suggest that PPG signals, particularly PRV, may correlate with cognitive function, providing insight into the autonomic nervous system’s role in cognitive health. PRV measures, which include metrics like the standard deviation of the pulse-to-pulse intervals (SDNN) and the root mean square of successive differences (RMSSD), have been shown to mirror heart rate variability (HRV) and thus offer a potential window into cardiovascular and autonomic functioning. Given that cardiovascular health and cognitive decline are interlinked, PPG may serve as a valuable tool in assessing cognitive function in aging populations [11].

Despite these promising developments, there remains a notable gap in the literature concerning the specific physiological characteristics of each cognitive domain measured by tools like the SNSB and how these might differ when assessed using PPG data. Existing research has primarily focused on general correlations between cognitive function and physiological signals, but little attention has been paid to domain-specific variations or the potential for using PPG as a screening tool.

Therefore, this study aims to fill this gap by analyzing the PRV data obtained from PPG measurements across different cognitive domains of the SNSB in the elderly. The traditional SNSB, while effective in assessing cognitive impairment, lacks the ability to observe physiological responses such as cardiovascular function. By exploring the potential of PPG data to be used as a physiological indicator for cognitive decline, this research sought to provide new insights into the association between cognitive and cardiovascular function.

Eventually, the study’s findings are expected to offer a novel approach to managing the health of the elderly, contributing to the development of strategies for health promotion and prevention by elucidating the complex interactions between cognitive function and cardiovascular health.

## 2. Materials and Methods

### 2.1. Data Collection

A total of 626 elderly women (mean age: 71.5 ± 6.26 years) participated in this study. The information of all participants is shown in Table 1. Prior to the clinical study, approval was obtained from the Chonnam National University Hospital Institutional Review Board (IRB: CNHU-2019-279), and informed consent was obtained from all participants after providing a detailed explanation of the experiment.

**Figure 1:**
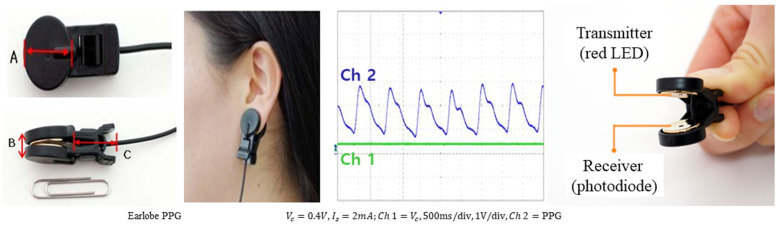
Wearable earlobe type PPG device and specification.

All participants were instructed to sit comfortably in a chair and maintain a stable state while a clip-type PPG (Model: EP520, LAXTHA Inc., Daejeon, Republic of Korea) was attached to their left earlobe for heart rate measurement. The measurement was conducted for a total of 5 min. The PPG device uses visible light with a wavelength of 640 nm, consisting of a transmitter that emits red LED light and a receiver that detects the light. The response frequency of the receiver is 0.3–5 Hz (Figure 1).

In addition to the PPG measurement, participants were assessed using SNSB-II, which evaluates various cognitive domains such as attention, language, visuospatial function, memory, and executive function.

### 2.2. Seoul Neuropsychological Screening Battery–Dementia Version

The Seoul Neuropsychological Screening Battery (SNSB) is a comprehensive neuropsychological assessment tool that is widely used in Korea and was developed by neuropsychologists in 2003. The SNSB is a standardized tool that can assess various cognitive functions and was later upgraded to SNSB-II. The SNSB-II improved the reliability and accuracy of cognitive function evaluation through standardized data and assessment tools. SNSB-II includes five major cognitive domains: attention, language and related functions, visuospatial functions, memory, and frontal/executive functions [5].

The development of SNSB-II led to the development of SNSB-D, the Seoul Neuropsychological Screening Battery–Dementia Version. The SNSB-D was designed to improve the limitations of the existing SNSB-II, particularly by reducing the assessment time and providing a Global Cognitive Function (GCF) score. For example, in the language and related function domain of SNSB, the Korean–Boston Naming Test (K-BNT) was simplified to achieve a more streamlined assessment. Instead of assessing all 60 subcategories such as spontaneous speech, auditory comprehension, repetition, reading, writing, and the four components of Gerstmann syndrome, limb and buccofacial praxis was excluded. This modification enabled efficient cognitive assessment in a shorter time than SNSB. SNSB-D is suitable for patients or participants with a reduced attention span through a short and focused assessment, and the GCF score can be assessed by summing the scores by each domain.

SNSB-D evaluates various cognitive domains, such as attention, language and related functions, visuospatial functions, and frontal/executive functions, with each domain score based on specific neuropsychological tests from the original SNSB-II. For example, the attention domain is evaluated using the Digit Span Test (DST), the language and related functions domain is evaluated using the Korean–Boston Naming Test (K-BNT) and Controlled Oral Word Association Test (COWAT), the visuospatial function domain is evaluated using the Rey Complex Figure Test (RCFT), and the frontal/executive functions domain is evaluated through the motor persistence test, the go-no-go test, and the verbal fluency test.

The SNSB-D provides a valid and reliable assessment tool for various levels of cognitive impairment, from normal cognition to mild cognitive impairment (MCI) and Alzheimer’s disease (AD). Notably, the GCF scores differed significantly among the normal cognition (NC), mild cognitive impairment (MCI), and Alzheimer’s disease (AD) groups (*p* < 0.001). Post hoc analyses revealed lower GCF scores for the AD group compared to the MCI group (*p* < 0.001) and for the MCI group compared to the NC group (*p* < 0.001). This highlights SNSB-D’s robustness in detecting cognitive decline, making it a crucial instrument for both clinical assessment and research. The GCF score and domain scores comprehensively assess cognitive functions and facilitate the long-term monitoring of dementia patients. In this study, we utilized the six domains of the SNSB-D, including the GCF for analysis [12].

### 2.3. Group Classification

In this study, we classified the participants into two groups based on the SNSB-D score. Following the results of previous studies, participants were divided into a High Cognitive Performance (HCP) group and a Low Cognitive Performance (LCP) group for each domain [12].

Specifically, for the first domain, attention, participants scoring 9 or higher were classified into the HCP group, while those scoring below 9 were classified into the LCP group. For the other domains, we used a cut off score of 23 points for language and related functions, 30 points for visuospatial function, 73 points for memory, 51 points for frontal/executive functions, and 200 points for Global Cognitive Function (GCF), which evaluates overall cognitive function.

The demographic information for each group is presented in Table 2. For accurate analysis, cases with any missing scores in the subtests were excluded from the analysis.

### 2.4. Data Analysis

#### 2.4.1. PPG Analysis

The PPG data were measured for a total 5 min at a sampling frequency of 250 Hz. From the recorded data, the first and last 1 min were excluded, and the remaining 3 min of data were used for analysis. MATLAB R2023b (MathWorks Inc., Natick, MA, USA) was used for signal processing and the analysis of the measured data.

##### Screening

Before analyzing the PPG data, a screening process was performed to ensure data reliability. Data that contained waveforms outside the measurement range due to external noise or motion, or that were not recorded due to technical issues such as poor contact with the PPG device sensor during measurement, were excluded. Each raw signal of the measured data was manually inspected and reviewed to identify and exclude such cases.

##### Preprocessing

To remove noise caused by movements outside the heart rate range, a bang-pass filter with a bandwidth of 0.4–4 Hz was applied [13]. Additionally, a trend filter was applied to remove trends and changes in the data [14].

##### Time Domain Feature Extraction

Time domain features were extracted and categorized as the mean and standard deviation of the Systolic-to-Systolic intervals (SDNN), heart rate (HR), standard deviation of the heart rate (SD_HR), root mean square of successive differences (RMSSD), NN50, and pNN50. The systolic-to-systolic interval was calculated by measuring the interval between each systolic peak, calculating the mean and standard deviation, and obtaining SDNN. In addition, the RMSSD was extracted by squaring the systolic-to-systolic intervals, calculating their mean, and taking the square root of that mean. NN50 was calculated by counting the number of systolic-to-systolic intervals that exceeded 50 ms, while pNN50 was determined as the proportion of NN50 intervals out of the total NN intervals. The mean heart rate was extracted by dividing the average systolic-to-systolic interval by 60, and its standard deviation was also calculated. Finally, the standard deviation of successive differences in the systolic-to-systolic intervals (SDSS) was extracted [15,16]. The time-domain features are explained in Table 3.

##### Frequency Domain Feature Extraction

Frequency domain feature extraction was performed by converting the systolic-to-systolic intervals into milliseconds and applying a Fast Fourier Transform (FFT). The frequency bands were divided into Low Frequency (LF, 0.04–0.15 Hz), and High Frequency (HF, 0.15–0.4 Hz), and the power for each band was calculated. The powers of LF and HF were then summed to obtain PW_LF and PW_HF, respectively. Additionally, the ratio of LF to HF (LF/HF) was calculated to assess the balance between sympathetic and parasympathetic nervous activity, as shown in Table 3.

Non-linear analysis features, such as SD1 and SD2, were also extracted, as illustrated in Table 3. SD1 reflects parasympathetic activity by using the standard deviation of the differences between successive heartbeats, while SD2 represents the overall balance of the autonomic nervous system using the standard deviation of their sum. The ratio of SD2 to SD1 (SD2/SD1) was also calculated [15,16].

#### 2.4.2. Statistical Analysis

##### Crude Analysis

The differences between the two groups were compared using time domain features, including the mean systolic-to-systolic interval, SDNN, HR, SD_HR, RMSSD, NN50, pNN50, peak value, and SDSS; frequency domain features, including power, PW_LF, PW_HF and LF/HF; and non-linear analysis features, including SD1, SD2, and SD2/SD1. To determine the significance of differences between the two groups, Welch’s *t*-test, a form of independent sample *t*-test, was used [17]. The *t*-test analysis was conducted using Python (version 3.11.5) in the Spyder (version 5.4.3) environment, utilizing the statsmodels package (version 0.14.0) and scipy (version 1.11.1) for statistical analysis. The significance level was set at 0.05, and *p*-values less than or equal to 0.05 were considered statistically significant.

##### Adjusted Analysis

In addition to differences in the PRV features between the two groups, there were significant differences in age and years of education. The HCP group was younger than the LCP group, which may be attributed to the impact of age on autonomic nervous system function [18]. Previous research also suggested that years of education can influence cognitive function test results [19]. To control for these confounding variables, the Generalized Linear Model (GLM) was used to adjust for age and years of education in the analysis of PRV features.

In the GLM analysis, regression was performed using Ordinary Least Squares (OLS) methods, which estimated the regression coefficients by minimizing residuals [20]. After adjusting for the effects of age and years of education, *T*-tests were performed on the PRV features. The analysis process and method were carried out using Python packages, and differences between the two groups were examined using Welch’s *t*-test [17]. In the adjusted analysis, the significance level was 0.05, and *p*-values less than or equal to 0.05 were considered statistically significant. This process ensured a more accurate and reliable determination of the differences between the groups.

## 3. Results

### 3.1. Feature Extraction

In this study, the PRV features of two groups were extracted for each domain, including attention, language and related functions, visuospatial function, memory, frontal/executive functions, and global cognitive function. Significant differences in these variables were observed based on age and years of education. Adjusted analysis was performed to consider these differences, and a comparison with the crude analysis results found that the effects of age and years of education were minimized. Differences in the mean values of the features before and after adjustment were observed, confirming that the adjustment effectively minimized the influence of age and years of education on the features. The values of features in each domain were shown in Table 4.

### 3.2. Statistical Analysis

*T*-tests were performed for each domain to compare the PRV features between the two groups. The analysis was divided into crude analysis, which did not consider age and years of education, and adjusted analysis, which adjusted for these variables. The features that showed significant differences (*p* < 0.05) between the two groups were as follows.

First, in the attention domain, a significant difference was found in HR in the crude analysis; however, this difference disappeared in the adjusted analysis. This suggests that age and years of education affected HR in the attention domain. In the language and related functions domain, a significant difference was observed in SD1 before adjustment, but this difference disappeared after adjustment. On the other hand, the LF/HF ratio did not show a significant difference before adjustment, but showed a significant difference after adjustment. In the visuospatial function domain, there was a significant difference in power and PW_LF in the crude analysis, but after adjustment, additional significant differences were observed in SDNN, SD_HR, SD2, SD2/SD1, and SDSS. In the memory domain, significant differences were found in LF/HF, SD1, and SD2/SD1 before and after adjustment. This means that the memory domain is affected by these features regardless of age and years of education. In the frontal/executive functions domain, significant differences were observed in SDNN, SD_HR, power, PW_LF, PW_HF, SD1, SD2, and SDSS before adjustment. However, after adjustment, the differences in PW_HF and SD1 disappeared, while significant differences remained for the other features. Finally, in the global cognitive function domain, which evaluated overall cognitive function, significant differences were found between the two groups in SDNN, SD_HR, NN50, pNN50, PW_HF, SD1, and SDSS before adjustment; however, the differences disappeared in SDNN and SD_HR after adjustment, and a significant difference was found in LF/HF after adjustment.

This study investigated where PRV features show significant differences between the HCP group and the LCP group in each cognitive domain assessed through the SNSB-D. The features were adjusted for age and years of education, and features that still showed significant differences could be evaluated as being related to cognitive function. The features that have significant differences after adjusting can be confirmed in Table 4.

## 4. Discussion

This study analyzed the differences in autonomic nervous system function between the HCP and LCP groups in each cognitive domain assessed by the SNSB-D. While there are many previous studies on the association between cognitive function and biosignals, there is lack of research investigating the relationship across specific cognitive domains [21,22,23]. Therefore, this study specifically examined the association between the individual cognitive domain score and the autonomic nervous system, demonstrating the importance of PRV feature analysis considering age and years of education.

First, we investigated the association between the autonomic nervous system and performances in the individual cognitive domain of attention, language and related function, visuospatial functions, memory, and frontal/executive functions, by comparing the high cognitive performance group (HCP) and the low cognitive performance group (LCP). The crude analysis showed that there were significant differences in age and years of education between the two groups. Additionally, based on previous studies indicating that aging affects autonomic nervous system function and that higher levels of education are associated with lower blood pressure, we determined that adjusted analysis was necessary to minimize the effects of these two variables [18,24]. As a result, we were able to identify the differences between the results of the crude and adjusted analyses. The results of the adjusted analysis, which minimized the influence of these two variables, were more reliable.

In the attention domain, the relationship between the autonomic nervous system and attention was not clear after adjusting for age and years of education. This suggested that there may not be a strong association between the autonomic nervous system and attention. In the language and related function domain, the LCP group had a significantly lower LF/HF ratio, which showed a correlation between language and parasympathetic nerve activation. According to a previous study, as the speaking speed increased, sympathetic nerve system activity increased and parasympathetic nervous system activity decreased [25]. Additionally, previous study results showed that learning is better when the sympathetic nervous system is more active among autonomic nervous system activities in language learning, and this result was similar to the results of this study [26]. In the visuospatial function domain, the LCP group showed significantly higher values in PRV features such as SDNN, PW_LF, and SDSS, which are associated with the autonomic nervous system’s response to external stress [16]. Generally, a higher ability to regulate the autonomic nervous system suggests a quicker response to external stimuli such as stress. However, since this study measured data during a 5 min rest period, it may have a different meaning in the LCP group. Higher values for these features even in a comfortable resting state suggest that this group might be more sensitive to stress or generally experience higher levels of anxiety [27]. Previous studies have also shown that individuals with depression or anxiety exhibit higher HRV values during rest, which can indicate increased vagal nerve tension due to heightened parasympathetic activity [28,29]. Therefore, it could be interpreted that the group with low visuospatial function is more sensitive to stress. In the memory domain, the LCP group had significantly higher values in features related to parasympathetic activity, indicating that the group with lower memory abilities had relatively higher parasympathetic activation [16]. In the frontal/executive function domain, similar to the visuospatial domain, the LCP group showed significantly higher values in PRV features associated with regulating the autonomic nervous system in response to stress. This suggests that the group with lower frontal/executive functions may be more sensitive to stress or in a state of anxiety [28,29]. Finally, in the global cognitive function (GCF) domain, the LCP group exhibited significantly higher values in PRV features related to parasympathetic activity [16]. These results are consistent with previous studies. A previous study comparing the correlation between cognitive function and the autonomic nervous system using 24 h ECG reported that people with higher cognitive function had more activated sympathetic nerves. This result is consistent with the results of this study, in which the LCP group showed reduced sympathetic nerve activation in the autonomic nervous system and relatively more parasympathetic nerve activation in the GCF domain, reflecting overall cognitive function. In addition, a study using cardiac ultrasound reported that people with early Alzheimer’s disease had a defect in sympathetic nervous system function. This result is also similar to the results of this study. Through comparison with previous studies, we confirmed the possibility of evaluating the association between the autonomic nervous system and cognitive function through PRV [30,31].

This study had some limitations. First, we did not consider the underlying comorbidities such as hypertension, mood disorder and diabetes that could affect the PRV results. Additionally, PRV features had only an indirect impact on cognitive function via autonomic nervous system and cardiovascular aging, and the interpretation of its results is limited. Despite these limitations, however, this study showed that PRV features could be useful indicators of cognitive decline, and it is expected that future research could contribute to developing cognitive function classification models or diagnostic models for Alzheimer’s disease, MCI, and other conditions using PRV variables.

## 5. Conclusions

In this study, we analyzed the differences in PRV features between groups with high and low cognitive function scores using SNSB-D. We showed that the autonomic nervous system, especially the parasympathetic nervous system, was more activated in the group with a low cognitive score. In particular, parasympathetic nerve activation was dominant. This suggests that the group with low cognitive function is more sensitive to external stimuli or stress in daily life, and that in individuals with low cognitive function, the sympathetic nerves in the autonomic nervous system are less activated, so the parasympathetic nerves are relatively more activated [32]. Future research should focus on simultaneously analyzing the correlation between brain activity, the autonomic nervous system, and cognitive function to establish a more direct relationship between PRV features and cognitive function. Such studies are expected to contribute to the early diagnosis and management of cognitive function disorders.

## Figures and Tables

**Table 1:** Participant information.

Characteristic	Value (Mean ± SD)
Age (year)	71.5 ± 6.26
Education (year)	9.12 ± 4.27
Height (cm)	153.14 ± 5.47
Weight (kg)	59.18 ± 20.75

**Table 2:** Demographic information for each domain group.

Domain (Cut Off Score)	Attention (9)		Language and Related Function (23)		Visuospatial Function (30)		Memory (73)		Frontal/ Executive Function (51)		GCF (200)	
Group	HCP	LCP	HCP	LCP	HCP	LCP	HCP	LCP	HCP	LCP	HCP	LCP
Subject	299	274	253	291	480	99	337	210	302	237	247	258
Score	10.68 ± 1.65	7.11 ± 0.85	24.35 ± 1.11	18.70 ± 3.29	34.27 ± 1.75	23.84 ± 6.07	91.30 ± 12.77	58.66 ± 10.64	58.81 ± 5.44	43.49 ± 5.76	222.96 ± 6.40	172.50 ± 8.91
Age (year)	*** 69.81 ± 6.10	*** 72.97 ± 5.83	*** 69.24 ± 5.92	*** 72.71 ± 3.63	*** 70.85 ± 6.28	*** 74.39 ± 5.29	*** 69.93 ± 6.01	*** 73.07 ± 5.91	*** 69.34 ± 6.06	*** 73.15 ± 5.39	*** 68.83 ± 5.90	*** 72.65 ± 5.66
Education (year)	*** 10.90 ± 3.66	*** 7.25 ± 3.78	*** 11.45 ± 3.33	*** 7.78 ± 3.63	*** 9.90 ± 3.80	*** 5.22 ± 3.72	*** 10.46 ± 3.90	*** 7.35 ± 3.65	*** 11.04 ± 3.56	*** 7.43 ± 3.66	*** 11.47 ± 3.45	*** 8.05 ± 3.44
Height (cm)	153.97 ± 5.47	152.40 ± 5.45	154.25 ± 5.39	152.63 ± 5.44	153.59 ± 5.47	150.92 ± 5.34	153.95 ± 5.36	152.16 ± 5.64	154.50 ± 5.22	151.80 ± 5.42	154.69 ± 5.36	152.22 ± 5.31
Weight (kg)	58.13 ± 8.27	58.84 ± 7.89	58.47 ± 7.82	58.34 ± 8.42	58.42 ± 8.12	58.18 ± 8.13	59.06 ± 7.89	57.73 ± 8.45	58.47 ± 7.52	58.29 ± 8.77	58.95 ± 7.57	57.87 ± 8.58

All values were defined as mean ± standard deviation, HCP: High Cognitive Performance, LCP: Low Cognitive Performance, GCF: Global Cognitive Function. Significant differences were indicated by *** (*p* < 0.001).

**Table 3:** Description of PRV features.

Features	Description	Unit
SDNN	Standard deviation of NN intervals	ms
RMSSD	Root mean square of successive RR interval differences	ms
SDSS	Standard deviation of the average NN intervals for each 5 min segment of a 24 h HRV recording	ms
NN50	The number of adjacent NN intervals that differ from each other by more than 50 ms	
pNN50	Percentage of successive RR intervals that differ by more than 50 ms	%
Total Power	The signal energy found within a frequency band	≤0.4 Hz
LF	Absolute power of the low frequency band	0.04–0.15 Hz
HF	Absolute power of the high frequency band	0.15–0.4 Hz
LF/HF	Ratio of LF to HF power	%
SD1	Poincare plot standard deviation perpendicular to the line of identity	ms
SD2	Poincare plot standard deviation along the line of identity	ms
SD2/SD1	Ratio of SD2 to SD1	%

**Table 4:** Comparison of PRV feature values between HCP and LCP of each cognitive domain, adjusted for age and years of education.

Domain	Attention		Language and Related Function		Visuospatial Function		Memory		Frontal/ Executive Function		GCF	
Group	HCP	LCP	HCP	LCP	HCP	LCP	HCP	LCP	HCP	LCP	HCP	LCP
Avg Systolic-to-Systolic Time (ms)	0.86 ± 0.09	0.87 ± 0.09	0.85 ± 0.09	0.84 ± 0.09	0.84 ± 0.09	0.85 ± 0.10	0.86 ± 0.09	0.85 ± 0.09	0.87 ± 0.09	0.87 ± 0.10	0.86 ± 0.09	0.86 ± 0.09
SDNN (s)	0.03 ± 0.07	0.03 ± 0.07	0.02 ± 0.07	0.02 ± 0.07	* 0.02 ± 0.07	* 0.03 ± 0.08	0.02 ± 0.07	0.02 ± 0.07	* 0.03 ± 0.07	* 0.04 ± 0.08	0.03 ± 0.06	0.04 ± 0.08
HR (beat/min)	70.14 ± 7.96	69.07 ± 7.62	71.01 ± 7.71	72.03 ± 7.84	71.30 ± 7.77	70.59 ± 7.98	70.11 ± 7.41	71.40 ± 8.14	69.72 ± 7.66	69.14 ± 7.85	69.94 ± 7.71	70.42 ± 7.70
SD_HR (beat/min)	1.63 ± 4.14	1.95 ± 4.29	0.92 ± 3.95	1.08 ± 4.43	* 1.01 ± 4.04	* 1.95 ± 5.04	1.01 ± 4.10	0.95 ± 4.41	* 1.61 ± 3.96	* 2.38 ± 4.52	1.57 ± 3.75	2.25 ± 4.51
RMSSD	857.05 ± 99.04	869.24 ± 99.12	843.54 ± 97.41	832.10 ± 100.09	840.05 ± 97.88	852.18 ± 105.94	857.52 ± 96.38	843.69 ± 99.50	863.48 ± 95.19	872.95 ± 101.15	861.80 ± 97.91	857.38 ± 96.85
NN50	61.78 ± 88.66	68.14 ± 87.30	53.62 ± 86.30	62.03 ± 92.25	47.81 ± 89.30	44.88 ± 84.23	47.80 ± 85.14	51.43 ± 92.71	67.29 ± 88.61	79.83 ± 89.67	* 68.62 ± 78.70	* 88.31 ± 97.89
pNN50	18.23 ± 20.20	19.49 ± 19.88	15.71 ± 19.35	17.91 ± 21.30	14.14 ± 20.44	13.13 ± 18.95	14.66 ± 19.33	15.33 ± 21.25	19.68 ± 20.20	22.20 ± 20.36	* 19.79 ± 18.03	* 23.79 ± 22.13
Peak_value	859.65 ± 94.12	871.35 ± 94.32	848.32 ± 92.81	836.06 ± 94.71	844.42 ± 93.29	853.69 ± 99.75	861.92 ± 91.48	847.81 ± 94.73	866.05 ± 90.89	873.82 ± 95.49	864.38 ± 93.61	857.85 ± 91.68
Power	1055.25 ± 324.42	1098.79 ± 340.92	993.75 ± 322.22	981.90 ± 339.44	* 1011.57 ± 317.78	* 1095.22 ± 397.23	1044.88 ± 332.44	1012.72 ± 329.31	* 1062.70 ± 318.90	* 1120.80 ± 347.03	1050.38 ± 313.98	1074.24 ± 337.35
PW_LF	79.33 ± 74.58	88.48 ± 77.90	65.97 ± 76.17	62.34 ± 72.65	** 76.04 ± 70.63	** 101.34 ± 96.30	84.08 ± 80.13	74.20 ± 68.30	* 77.84 ± 74.08	* 92.15 ± 78.68	72.74 ± 72.02	78.86 ± 75.91
PW_HF	72.18 ± 137.99	85.80 ± 150.14	45.41 ± 131.40	59.22 ± 156.07	43.78 ± 140.30	68.05 ± 168.66	43.65 ± 140.56	49.27 ± 154.12	80.30 ± 137.41	104.86 ± 153.81	* 73.42 ± 129.47	* 98.74 ± 53.47
LF/HF	0.81 ± 0.27	0.81 ± 0.28	* 0.79 ± 0.27	* 0.74 ± 0.28	* 0.84 ± 0.28	* 0.87 ± 0.28	* 0.85 ± 0.27	* 0.79 ± 0.29	0.73 ± 0.27	0.73 ± 0.28	* 0.72 ± 0.27	* 0.66 ± 0.28
SD1	−0.01 ± 0.06	−0.01 ± 0.06	−0.02 ± 0.05	−0.01 ± 0.06	−0.02 ± 0.06	−0.02 ± 0.07	* −0.03 ± 0.05	* −0.018 ± 0.07	−0.01 ± 0.05	0.00 ± 0.06	** −0.01 ± 0.04	** 0.01 ± 0.07
SD2	0.04 ± 0.09	0.05 ± 0.09	0.03 ± 0.08	0.03 ± 0.09	* 0.04 ± 0.08	* 0.06 ± 0.10	0.04 ± 0.09	0.03 ± 0.08	* 0.04 ± 0.08	* 0.06 ± 0.09	0.04 ± 0.08	0.05 ± 0.09
SD2/SD1	4.95 ± 2.28	5.18 ± 2.50	4.65 ± 2.42	4.47 ± 2.33	* 5.11 ± 2.32	* 5.65 ± 2.60	** 4.96 ± 2.63	** 4.39 ± 1.89	4.33 ± 2.19	4.49 ± 2.56	4.20 ± 2.27	4.01 ± 2.50
SDSS	0.02 ± 0.07	0.03 ± 0.07	0.016 ± 0.07	0.02 ± 0.07	* 0.02 ± 0.07	* 0.03 ± 0.08	0.02 ± 0.07	0.02 ± 0.07	* 0.03 ± 0.07	* 0.04 ± 0.08	0.027 ± 0.06	0.04 ± 0.08

All values were defined as mean ± standard deviation, and significant differences were indicated by * (*p* < 0.05), ** (*p* < 0.01).

## Data Availability

The data presented in this study are available upon request from the corresponding author.
